# How to minimize adverse effects of physical workplace violence on health sector workers: A preliminary study

**DOI:** 10.3389/fpsyg.2022.998856

**Published:** 2022-10-28

**Authors:** Jingjing Lu, Jingjing Cai, Wenchen Shao, Zhaocheng Wang

**Affiliations:** ^1^School of Public Health, School of Medicine, Zhejiang University, Hangzhou, Zhejiang, China; ^2^Department of Anatomy and Cell Biology, McGill University, Montreal, QC, Canada

**Keywords:** health sector worker, workplace violence, physical violence, medical institution, mental health

## Abstract

**Purpose:**

This paper is an exploratory study to investigate possible remedial measures accounting for a relatively favorable prognosis of health sector workers who have experienced physical WPV in Zhejiang province, China.

**Methods:**

Following a proportionate stratified sampling strategy, five tertiary hospitals (in the developed capital city of Hangzhou and other prefecture-level cities), eight secondary hospitals (in counties), and thirty-two primary care facilities (16 urban community health centers and 16 rural township health centers) were conveniently selected. Among 4,862 valid respondents out of 6,089 self-conducted questionnaires, 224 health sector workers who have been directly exposed to physical WPV in the past year were included in the present study.

**Results:**

The present study has three major findings: (1) Victims’ satisfaction with the resolution of the physical WPV conflict was directly associated with the favorable prognosis. (2) Taking days off from work after the violence can promote victims’ satisfaction with the resolution of the physical WPV conflict. (3) Knowing that relevant departments investigated the case can promote victims’ satisfaction with the resolution of the physical WPV conflict.

**Conclusion:**

We propose a combined gesture of “offering adequate days off work after physical WPV” and “every physical violence must be investigated” that should be taken by all medical institutions in China. Health sector workers who get involved in physical WPV incidents should prioritize their safety and avoid any behavior that may intensify the conflicts.

## Introduction

Workplace violence (WPV) is defined as “incidents where staff is abused, threatened or assaulted in circumstances related to their work, involving an explicit or implicit challenge to their safety, well-being, or health” (Wynne et al., 1997), including physical and verbal violence, psychological abuse, sexual harassment and assault, and homicide (Di Martino, 2002). Being recognized as a global public health problem, WPV has affected the health sector disproportionately and become a major problem (Phillips, 2016). Health sector workers include doctors, nurses, and other staff who may be in direct contact with patients and visitors. According to one meta-analysis of 331,544 health sector workers from all over the world (Liu et al., 2019), 61.9% of these participants were exposed to any form of WPV, and 24.4% of them experienced physical violence in the past year.

The prevalence of WPV varies across nations and regions. For example, the prevalence of WPV in the health sector was reported to be higher in Asian countries than in European countries (Liu et al., 2019). Possible reasons were generated to account for this difference, including healthcare systems, numbers of health sector workers per 1,000 population, and government health expenditure in the health sector (Bandara, 2006). Yang and her colleagues’ study showed that differences in culturally afforded scapegoating can also influence patients’ violence against health sector workers (Yang et al., 2021).

The detrimental effects of WPV on health sector workers have been well reported. According to a recent systematic review (Mento et al., 2020), reduced quality of life seems to be a general consequence of WPV among physicians and nurses. Exposure to WPV, either physically or verbally, was associated with depression (Wang et al., 2021), declined work enthusiasm (Li et al., 2017), and high levels of stress (Rayan et al., 2019) in health sector workers. Additionally, one study conducted in India pointed out the fact that exposure to WPV can not only exert negative effects on doctors but also influence doctors’ patient management (Kaur et al., 2020). Moreover, the widespread WPV against health sector workers can also make the general public harder to access primary health care resources. WPV can boost health sector workers’ intention to quit the profession, especially in developing countries, which may exacerbate the shortage of medical professionals (Kaur et al., 2020). All these consequences of WPV can eventually lead to a decrease in the productivity and quality of medical care and subsequently cause damage to health equality for the general public.

China has been no exception to this terrible torment. As noted by a national study conducted by the Chinese Hospital Association among 316 hospitals from different levels (Yao et al., 2014), the proportion of WPV against health sector workers rose from 90% in 2008 to 96% in 2012 with the proportion of physical WPV increased from 47.7% in 2008 to 67.7% in 2012. And other two regional studies (Zhang et al., 2018; Duan et al., 2019)showed that about 66 to 75% of health sector workers experienced different forms of WPV in the past year. Due to the high prevalence of WPV in the health sector, medical professionals in China have been considered as working in high-risk environments in recent decades (Xing et al., 2016; Guan, 2017). In one meta-analysis, it was reported that the estimated prevalence of physical violence, psychological violence, verbal abuse, threats and sexual harassment were 13.7, 50.8, 61.2, 39.4, and 6.3%, respectively, among Chinese health-care professionals (Lu et al., 2020). The prevalence of WPV in China varies from province to province, from hospital to hospital, and from department to department (Ma et al., 2021). A recent survey noted that about two thirds of Chinese physicians experienced doctor-patient conflicts, and in 2018, 3,308 people were prosecuted for intentionally harming medical personnel and causing disturbances in hospitals (Zhang et al., 2021).

Among different forms of WPV, physical WPV usually happens suddenly and can cause serious damages immediately. As noted, the vast majority of cases of serious WPV reported by mass media in China were physical in nature and were often committed with weapons (Ma et al., 2021). A growing number of researchers propose that Chinese legislation system should include the acts of injuring and killing health sector workers into the statutory aggravation of the crimes of intentional injury and intentional homicide to protect health sector workers’ rights (Xie and Wang, 2022). Interventions to prevent WPV against health sector workers can be divided into primary prevention, secondary prevention, and tertiary prevention (Hall et al., 2018). Primary prevention focuses on structural and social factors that may reduce WPV, for example, efforts from legislation system. Secondary prevention involves early actions to avoid the onset or worsening of mental health problems among victims of WPV. Tertiary prevention ensures victims of WPV can get timely and proper care and report the incident to relative authorities. We should realize that there are certain remedial measures, which prevent victims from getting severely mentally affected by the WPV incident, that medical institutions can adopt from the perspective of secondary and tertiary prevention. While it is urgent to shied health sector workers from physical WPV, comforting victims in time and helping them heal from trauma as quickly as possible are also significantly important. However, no empirical studies across multiple medical institutions focusing on secondary and tertiary prevention of physical WPV of health sector workers in China can be identified. This paper is an exploratory study to investigate possible remedial measures accounting for a relatively favorable prognosis of victims of physical WPV in medical institutions in Zhejiang province, China.

## Materials and methods

This study was a cross-sectional survey to explore possible remedial measures that could be taken by medical institutions after their employees experienced physical WPV in Zhejiang Province, China.

### Data collection

This survey was conducted from July 2016 to July 2017. A proportionate stratified sampling strategy was adopted to cover health sector workers from tertiary hospitals, secondary hospitals, and primary care facilities in both urban and rural areas. Five tertiary hospitals (in the developed capital city of Hangzhou and other prefecture-level cities), eight secondary hospitals (in counties), and thirty-two primary care facilities (16 urban community health centers and 16 rural township health centers) were conveniently selected. Then, in each facility, one specified administrator was designated as the research assistant to perform the survey after permissions from hospital managers or primary care facility directors was obtained. On the days of the survey, all health workers on duty were invited to participate with the help of these research assistants. A statement of consent was offered on the first page of the questionnaire. Participants were informed that this survey was fully voluntary, and anonymity was assured.

We distributed 6,089 questionnaires in total, of which 5,145 were completed. Then, 4,862 of them were identified as valid, representing a valid response rate of 79.8%. Among them, 224 respondents experienced physical workplace violence in the past year and were subsequently included in this study for further analysis.

### Study variables

This questionnaire comprised five sections: (1) Social demographic characteristics and professional background of health sector workers. (2) Individual responses during the incident. (3) Institutional attitudes and responses toward the incident. (4) Aftermaths of the incident. (5) Impacts of the incident on health sector workers.

Social demographic characteristics and professional background of health sector workers included gender, age, level of hospitals, types, and the title of health workers.

The impact of the incident on health sector workers was assessed with eight items describing different types of negative prognoses victims may have after the exposure to WPV.

Possible remedial measures accounting for a relatively favorable prognosis of victims of physical WPV in medical institutions, which were extracted from our previous qualitative interviews with health sector workers (unpublished), included (1) Does the medical institution you are working for encourage tolerating violence? (2) Did the medical institution you are working at offer you days off work after the violence? (3) Was the case investigated by relevant departments? (4) Were the perpetrators punished in any way? (5) Were you satisfied with how the incident was handled?

### Ethics

The ethics committee of our university reviewed and approved this study. We informed participants that the survey was voluntary and obtained verbal consent before the commencement of the survey.

### Data analysis

A latent class analysis (LCA) was conducted among health sector workers who experienced physical workplace violence in the past year (*n* = 224) based on the impact of the incident on them using Mplus Version 8.3. LCA creates groups based on the similarity of participants’ patterns of responses to a set of dichotomous variables. To identify the optimal number of classes, we examined the Bayesian information criterion (BIC), Lo–Mendell–Rubin (LMR) likelihood ratio test, and entropy. A model is considered as fitting with a relatively lower BIC, a value of *p* <0.05 of the LMR, and relatively higher entropy. Optimal models were chosen based on goodness of fit and parsimony (Table 1).

**Table 1:** Model fit statistics for each of the fitted latent class analysis models.

Model	BIC	Entropy	LMR	Prevalence *N*(%)				
				1	2	3	4	5
1	2018.165			224(100.0)				
2	1733.737	0.897	366.778^***^	50(22.32)	174(77.68)			
3	1760.001	0.931	59.634^***^	174(77.68)	1(0.45)	49(21.88)		
4	1793.703	0.887	52.281^*^	154(68.75)	1(0.45)	22(9.82)	47(20.98)	
5	1859.360	0.832	20.690	57(25.45)	19(8.48)	1(4.45)	22(9.82)	125(55.80)

* *p* < 0.05;

*** *p *< 0.001.

After the optimal model was identified from the LCA, we stratified participants into two groups: 1) the slightly affected and 2) the severely affected (Table 2). Then, participants’ social demographic characteristics were compared using Chi-squared tests and t-tests (Table 3). Binary logistic regression models were applied to examine the associations between the impact of physical WPV incidents on health sector workers and potential remedial measures. Analyses were adjusted for gender, age, level of hospitals, types, and the title of health workers. All analyses were performed using SPSS 20.0 version and assumed a statistical significance level of *p* < 0.05.

**Table 2:** Impacts of workplace violence on health workers stratified by group *N*(%).

	Slightly affected (*N* = 50)	Severely affected (*N* = 174)	*p*
*Repeated, disturbing memories, thoughts, or images of the attack*			<0.001
Yes	13(7.69)	156(92.31)	
No	37(67.27)	18(32.73)	
*Made you trust less in patients as a whole*			<0.001
Yes	9(5.59)	152(94.41)	
No	41(65.08)	22(34.92)	
*Avoiding thinking about or talking about the attack*			<0.001
Yes	5(4.00)	120(96.00)	
No	45(45.45)	54(54.55)	
*Be more cautious when dealing with patients*			<0.001
Yes	30(14.71)	174(85.29)	
No	2(100.00)	0(0.00)	
*Being ‘super-alert’ or watchful and on guard when dealing with patients*			<0.001
Yes	27(13.43)	174(86.57)	
No	23(100.00)	0(0.00)	
*Fearful of dealing with urgent or severe cases*			<0.001
Yes	7(4.29)	156(95.71)	
No	43(70.49)	18(29.51)	
*Often consider how to protect yourself while dealing with patients*			<0.001
Yes	25(13.23)	164(86.77)	
No	25(71.43)	10(28.57)	
*Wanting to quit the current post*			<0.001
Yes	5(3.82)	126(96.18)	
No	45(48.39)	48(51.61)	

**Table 3:** Characteristics and potential remedial measures of health workers who experienced workplace violence in the past year stratified by group *N*(%).

	Slightly affected (*N* = 50)	Severely affected (*N* = 174)	*p*
*Gender*			0.518
Male	24(24.24)	75(75.76)	
Female	25(20.33)	98(79.67)	
Age Mean(SD)	36.06(9.78)	34.57(8.09)	0.285
*Level of hospitals*			0.887
Tertiary hospitals	15(22.39)	52(77.61)	
Secondary hospitals	27(22.50)	93(77.50)	
Urban community health facilities	4(17.39)	19(82.61)	
Rural community health facilities	4(28.57)	10(71.43)	
*Types of health workers*			0.146
Doctors	22(18.64)	96(81.36)	
Nurses	18(23.08)	60(76.92)	
Others	10(35.71)	18(64.29)	
*Title*			0.408
Junior	18(19.15)	76(80.85)	
Intermediate	18(21.43)	66(78.57)	
Senior	11(33.33)	22(66.67)	
Other	3(23.08)	10(76.92)	
*Does the medical institution you are working for encourage tolerating violence?*			0.737
No	19(24.05)	60(75.95)	
Yes/ I don’t know	31(21.38)	114(78.62)	
*Did the medical institution you are working offer you days off work after the violence?*			1.000
Yes	10(21.74)	36(78.26)	
No	40(22.47)	138(77.53)	
*Was the case investigated by relevant departments?*			0.459
Yes	10(17.86)	46(82.14)	
No/ I don’t know	40(23.81)	128(76.19)	
*Were the perpetrators punished in any way?*			0.132
Yes	16(30.19)	37(69.81)	
No	34(19.88)	137(80.12)	
*Were you satisfied with how the incident was handled?*			0.005
Yes	24(34.29)	46(65.71)	
No	26(16.88)	128(83.12)	

## Results

Correlates of class membership were investigated with the optimal number of classes identified (Table 1). Both the Model 2 and the Model 3 showed a relatively lower BIC with a value of p of the LMR lower than 0.05 and a relatively higher entropy. However, one class in the Model 3 only accounted for 0.45 0f the total sample (1 individual), which might not provide reliable estimates of class-specific parameters in later analyses. Therefore, we chose the Model 2 as the final model.

Descriptive statistics of each class and comparisons are summarized in Table 2.

Table 3 shows differences in participants’ social demographic and professional background characteristics between the slightly affected and the severely affected group. These two groups didn’t show any differences in gender, age, level of hospitals, types, and the title of health workers.

As Table 4 shows, with adjustments to gender, age, level of hospitals, types, and the title of health workers, victims who were not satisfied with how the incident was handled were more likely to be severely affected by the physical WPV incident (OR = 3.50, 95%CI = (1.59, 7.69), *p* = 0.002).

**Table 4:** Regression coefficients for the impact of physical WPV on health sector workers and potential remedial measures with adjustment for social demographic characteristics.

	Severely affected		*p*
	OR	95%CI	
*Does the medical institution you are working for encourage tolerating violence?*			
No	1.00		
Yes/ I don’t know	1.02	(0.44,2.31)	0.978
*Did the medical institution you are working offer you days off work after the violence?*			
Yes	1.00		
No	1.32	(0.49,3.55)	0.583
*Was the case investigated by relevant departments?*			
Yes	1.00		
No/ I don’t know	0.56	(0.23,1.39)	0.213
*Were the perpetrators punished in any way?*			
Yes	1.00		
No	0.54	(0.22,1.34)	0.184
*Were you satisfied with how the incident was handled?*			
Yes	1.00		
No	3.50	(1.59,7.69)	0.002

Adjusted with gender, age, level of hospitals, types, and the title of health workers.

As Table 5 shows, with adjustments with gender, age, level of hospitals, types, and the title of health workers, victims who didn’t take days off after the violence (OR = 0.39, 95%CI = (0.17, 0.88), *p* = 0.024) and whose cases were not investigated by relevant departments (OR = 0.45, 95%CI = (0.21, 0.95), *p* = 0.036) were less likely to be satisfied with how the incident was handled.

**Table 5:** Regression coefficients for the health sector workers’ satisfaction and potential remedial measures with adjustment for social demographic characteristics.

	Health sector workers’ satisfaction		*p*
	OR	95%CI	
*Does the medical institution you are working for encourage tolerating violence?*			
No	1.00		
Yes/ I don’t know	0.91	(0.43,1.92)	0.803
*Did the medical institution you are working offer you days off work after the violence?*			
Yes	1.00		
No	0.39	(0.17,0.88)	0.024
*Was the case investigated by relevant departments?*			
Yes	1.00		
No/ I don’t know	0.45	(0.21,0.95)	0.036
*Were the perpetrators punished in any way?*			
Yes	1.00		
No	0.64	(0.28,1.48)	0.293

Adjusted with gender, age, level of hospitals, types, and the title of health worker.

## Discussion

This study is an exploratory study to investigate possible remedial measures accounting for a relatively favorable prognosis for victims of physical workplace violence against health sector workers. We have three major findings: (1) Victims’ satisfaction with the resolution of the physical WPV conflict was directly associated with the favorable prognosis. (2) Taking days off from work after the violence can promote victims’ satisfaction with the resolution of the physical WPV conflict. (3) Knowing that the case was investigated by relevant departments can promote victims’ satisfaction with the resolution of the physical WPV conflict.

Based on binary logistic analyses, this study proposed that medical institutions should offer health sector workers, who are the victims of physical WPV conflicts, days off from work to promote their satisfaction with the resolution of the physical WPV conflict, which may lead to a favorable prognosis for those victims. As noted, psychological distress is a substantial contributor to work re-entry efforts for victims of WPV (de Koning et al., 2017). And days off work will allow those victims to seek professional help to meet their unique and specific psychological needs. It is recommended that an early de-briefing model employing counselors with specialist skills can minimize the psychological effects following a WPV incident (Caldwell, 2006). Based on a randomized controlled explorative and comparative study, Tarquinio and his colleagues (Tarquinio et al., 2016) found that Shapiro’s Eye Movement Desensitization and Repreprocessing Recent Events Protocol (EMDR-RE; Shapiro, 2001) and delayed EMDR-RE can protect victims of WPV from PTSD symptoms. Additionally, tabletop scenario exercises can provide a context specific approach at low cost to improving medical institutions’ ability to correctly assess the risks of WPV and the comprehensiveness of the response (Brunero et al., 2021). While most tertiary and secondary medical institutions in China have set specialized departments to collect and process complaints of patients, it is frustrating that little has been done for their employees.

This study also noted that it is important to let those victims know that the case was investigated by relevant departments. Defending their rights after the violence when they may still be shocked, angry, and frustrated is a torment for those victims of WPV against health sector workers. The majority of health sector workers in China chose to be silent after the WPV, considering it’s fruitless to report the incident (Wang et al., 2018). In the present study, only 56 out of 224 health sector workers reported that their cases were investigated. In China, it is an alarming fact that law enforcement professionals and administrators of medical institutions generally tend to treat WPV against health sector workers as doctor-patient disputes rather than criminal behaviors. Those perpetrators were regarded as the weak and vulnerable side due to the information asymmetry by law enforcement and the general public (Yao et al., 2014). Most of these incidents were never seriously investigated (Zheng et al., 2007), let alone valid punishment of the perpetrators by law enforcement professionals.

Counterintuitively, while the case investigation was associated with victims’ satisfaction with the resolution of the physical WPV conflict, whether or not the perpetrators got punished showed no correlations with the satisfaction A possible explanation is that these victims regarded the case investigation as a closure of the incident; and their good nature as health sector workers makes them forgive those perpetrators. However, the impunity of perpetrators won’t help in controlling WPV against health sector workers. As international experience suggests, we should ensure that there are laws to abide by; those laws are observed and strictly enforced; and those lawbreakers are prosecuted to contain the widespread of WPV against health sector workers (Sun et al., 2016). Instead of being considered as legal rights protection by health sector workers, those laws should be considered as warnings to potential perpetrators.

Attacks from organizational outsiders like patients are unpredictable (Grandey et al., 2007). Aside from preventing WPV incidents, it is equally important for healthcare professionals to know how to react to a WPV incident. One of the important things for the safety of health sector workers during a WPV incident is to avoid intensification of the conflict. Behaviors like fighting back and swearing can lead the incident to a more unwanted violent situation, which may increase the odds of getting physical injures. One recent systematic review (Raveel and Schoenmakers, 2019) suggested that health sector workers should stay calm and apply de-escalation techniques to guarantee their safety during a WPV incident. De-escalation generally takes the form of a verbal loop (Price and Baker, 2012) and may not be suitable for physical workplace violence settings, especially when the patients are furious. When the de-escalation is not effective, as suggested by this review, health sector workers may better go away and take self-defense techniques or call security staff for help.

A public health crisis such as coronavirus disease 2019 (COVID-19) can influence the relationship between health sector workers and patients rapidly. The media’s reports and praise of health sector workers who fight against the COVID-19 enhanced the public’s support of health sector workers, which in turn can improve the relationship between health sector workers and patients (Zhou et al., 2021). Thus, we may infer that health sector workers may feel more upset based on their mixed experience of fighting against the COVID-19 and suffering from patients’ violence. With the layout and construction of normalized nucleic acid test sites in communities, we should pay extra attention to the well-being of health sector workers working at nucleic acid test site overtime.

Several limitations should be considered when interpreting these findings. First, the present study only included 224 health sector workers who experienced physical violence in the past year out of the 4,862 valid questionnaires. While the findings from the present study can provide new insights for health sector workers and medical institutions, these findings should be interpreted with caution, considering the limited generalizability. Second, the present study only involved health sector workers. Data from other direct key stakeholders, including medical institution managers, law enforcement agencies, and security staff can greatly enrich the present study. Third, the data collection date back to 6 years ago and by no means can we ignore the effects of the COVID-19 pandemic on doctor-patient relationships. Although it may be impossible for us to trace those every health sector worker who participated the current study, we are able to survey all these medical institutions enrolled in the present study again to explore potential changes and mechanisms.

## Conclusion

The present study explored possible remedial measures accounting for a relatively favorable prognosis of health sector workers who have experienced physical WPV. Based on our data analysis, we propose a combined gesture of “offering adequate days off work after physical WPV” and “every physical violence must be investigated” that should be taken by all medical institutions in China. Health sector workers who get involved in physical WPV incidents should prioritize their safety and avoid any behavior that may intensify the conflicts.

## Data availability statement

The data-sets analyzed in this study are available from the corresponding author on reasonable request.

## Ethics statement

The studies involving human participants were reviewed and approved by the ethics committee of our university. The patients/participants provided their written informed consent to participate in this study.

## Author contributions

JL: analyzed and interpreted the data, drafted the manuscript, and participated in the coordination of the study. ZW: participated in the coordination of the study, participated in critical review of the manuscript, and participated in the conception and design of the study. JC: drafted the manuscript and participated in the coordination of the study. WS: participated in critical review of the manuscript and participated in the conception and design of the study. All authors contributed to the article and approved the submitted version.

## Conflict of interest

The authors declare that the research was conducted in the absence of any commercial or financial relationships that could be construed as a potential conflict of interest.

## Publisher’s note

All claims expressed in this article are solely those of the authors and do not necessarily represent those of their affiliated organizations, or those of the publisher, the editors and the reviewers. Any product that may be evaluated in this article, or claim that may be made by its manufacturer, is not guaranteed or endorsed by the publisher.
